# Lupus anticoagulant hypoprothrombinemia syndrome in Bence-Jones protein κ-type multiple myeloma patient with phosphatidylserine-dependent antiprothrombin antibody

**DOI:** 10.1007/s00277-012-1600-5

**Published:** 2012-10-18

**Authors:** Yoshitaka Hara, Masanori Makita, Tatsunori Ishikawa, Kyosuke Saeki, Kazuhiko Yamamoto, Kenji Imajo, Midori Shima, Masahiro Ieko

**Affiliations:** 1 Department of Hematology and Oncology, Okayama City Hospital, 6-10 Amase Kita-ku, Okayama, 700-8557 Japan; 2 Department of Pediatrics, Nara Medical University, Kashihara, Japan; 3 Department of Internal Medicine, School of Dentistry, Health Sciences University of Hokkaido, Tobetsu, Japan

Dear Editor,

Approximately 2 % of multiple myeloma (MM) patients present with hemorrhage at diagnosis. However, hemorrhage due to abnormalities in the coagulation system is a rare complication [1, 2]. Although lupus anticoagulant (LA), which is infrequently reported to be in association with MM, is commonly a risk factor for arterial or venous thrombosis, bleeding tendencies in patients with LA are strongly related to a low prothrombin activity [3–6]. Acquired hypoprothrombinemia with LA, also called LA hypoprothrombinemia syndrome (LAHPS), is a rare disease which appears mostly in young females with systemic lupus erythematosus or in healthy children after viral infection and is usually associated with the presence of antiprothrombin antibodies [7]. We herein report the case of an 86-year-old male with Bence-Jones protein (BJP) κ-type MM who presented with hypoprothrombinemia and LA associated with antibodies directed to the phosphatidylserine–prothrombin complex (or phosphatidylserine-dependent antiprothrombin antibodies, aPS/PT). The patient was admitted with anemia and had no past history of bleeding disorders or thrombotic events. A urinalysis showed massive proteinuria (5.3 g/day), which was determined to be κ-type BJP using immunoelectrophoresis. Bone marrow aspiration showed proliferation of abnormal plasma cells. Computerized tomography showed hematomas in the bilateral gluteus maximus muscle and the supraclavicular area. Initial coagulation tests showed prolonged prothrombin time and activated partial thromboplastin time (aPTT) (Table 1). Reduced clotting activity of factors II (FII), VIII (FVIII), and IX (FIX) was noted in a pattern typical of that observed in previously reported cases of LAHPS [8, 9]. FVIII and FIX inhibitors were not detected. The prolonged aPTT with LA-sensitive aPTT reagent (PTT-LA Roche Diagnostics, Tokyo, Japan), which could not be corrected by mixing with normal plasma, suggested the presence of LA. The results were confirmed using the Staclot LA® assay, and the dilute Russell viper venom time test was used to confirm the presence of LA with the phospholipid-neutralizing LA test (Gradipore, Frenchs Forest, Australia). IgG/M anticardiolipin antibodies and IgG aPS/PT were negative, while strong positive IgM aPS/PT was detected, which was measured with ELISA using the phosphatidylserine–prothrombin complex as antigen immobilized on ELISA plates in the presence of CaCl_2_ [10]. Based on these findings, the patient was diagnosed as MM with LAHPS associated with aPS/PT and treated with melphalan and prednisolone (MP) therapy. The FII levels were observed to normalize after one cycle of MP therapy and the patient has remained in remission without any hemorrhage for 10 months.

**Table 1:** Laboratory findings

	MP therapy			Normal values
	Before	After		
		3 courses	6 courses	
PT (%)	26	56	64	70–130
aPTT (s)	65.3	49.0	44.6	25–38
Fibrinogen (mg/dl)	284			200–400
Factor II (%)	49	93	94	74–149
Factor V (%)	129			70–152
Factor VII (%)	69			63–143
Factor VIII (%)	43	33	43	62–145
Factor IX (%)	9	7	9	74–149
Factor X (%)	74			71–128
Factor XIII (%)	100			>70
LA test				
PTT-LA (s)	158.7			<48.2
Staclot LA (s)	58.0		58.1	<8.0
dRVVT ratio	3.0		2.67	<1.3
Anti-b2GPI (U)				
IgM	0			<29.8
IgG	6.3			<10.4
aPS/PT (U)				
IgM	>100		20.8	<13.0
IgG	0		0	<2.0
BJP	Positive	Negative	Negative	Negative
Plasma cell (%)	54.2	2.0	2.8	<3.5

*PT* prothrombin time, *dRVVT* dilute Russel’s viper venom time

In our case, aPTT continued to be prolonged with reduced levels of FVIII and FIX in spite of normalizing the FII level after therapy. LA and IgM aPS/PT remained positive, although these values were improved, suggesting that the presence of LA might have an influence on coagulation tests after treatment. There are very rare reports showing the presence of aPS/PT in patients with LAHPS [9]. These reports describe the patients as having bleeding tendencies with mildly reduced FII levels, similar to that observed in our patient. However, in previously reported child cases of LAHPS, severe hemorrhage usually occurs when the FII levels are very low (under 10∼15 %). It is possible that other coagulation factors associated with aPS/PT in LAHPS might be present. A diagnosis of LAHPS should always be considered in MM patients with bleeding tendencies associated with LA, and aPS/PT detection should be performed in conjunction with LA tests.
